# Carbonic anhydrase IX, a hypoxia-induced catalytic component of the pH regulating machinery in tumors

**DOI:** 10.3389/fphys.2013.00400

**Published:** 2014-01-08

**Authors:** Olga Sedlakova, Eliska Svastova, Martina Takacova, Juraj Kopacek, Jaromir Pastorek, Silvia Pastorekova

**Affiliations:** Department of Molecular Medicine, Institute of Virology, Slovak Academy of SciencesBratislava, Slovakia

**Keywords:** carbonic anhydrase IX, hypoxia, pH regulation, tumor microenvironment

## Abstract

Acidic tissue microenvironment contributes to tumor progression via multiple effects including the activation of angiogenic factors and proteases, reduced cell-cell adhesion, increased migration and invasion, etc. In addition, intratumoral acidosis can influence the uptake of anticancer drugs and modulate the response of tumors to conventional therapy. Acidification of the tumor microenvironment often develops due to hypoxia-triggered oncogenic metabolism, which leads to the extensive production of lactate, protons, and carbon dioxide. In order to avoid intracellular accumulation of the acidic metabolic products, which is incompatible with the survival and proliferation, tumor cells activate molecular machinery that regulates pH by driving transmembrane inside-out and outside-in ion fluxes. Carbonic anhydrase IX (CA IX) is a hypoxia-induced catalytic component of the bicarbonate import arm of this machinery. Through its catalytic activity, CA IX directly participates in many acidosis-induced features of tumor phenotype as demonstrated by manipulating its expression and/or by *in vitro* mutagenesis. CA IX can function as a survival factor protecting tumor cells from hypoxia and acidosis, as a pro-migratory factor facilitating cell movement and invasion, as a signaling molecule transducing extracellular signals to intracellular pathways (including major signaling and metabolic cascades) and converting intracellular signals to extracellular effects on adhesion, proteolysis, and other processes. These functional implications of CA IX in cancer are supported by numerous clinical studies demonstrating the association of CA IX with various clinical correlates and markers of aggressive tumor behavior. Although our understanding of the many faces of CA IX is still incomplete, existing knowledge supports the view that CA IX is a biologically and clinically relevant molecule, exploitable in anticancer strategies aimed at targeting adaptive responses to hypoxia and/or acidosis.

## Hypoxia and acidosis: hand-in-hand toward tumor progression

During solid tumor growth, subpopulations of cancer and stromal cells are exposed to variable conditions depending on the local and temporal supply of oxygen, nutrients, growth factors and signaling molecules by aberrant vasculature as well as on spatial constraints imposed by the surrounding normal tissue, elevated interstitial pressure, gradients of metabolic waste and other factors. These physiological conditions, together with variable genetic backgrounds of tumor cells caused by oncogenic events, result in the development of highly heterogeneous tumor tissue with a microenvironment characterized, among other features, by regions of hypoxia and/or acidosis (Fang et al., 2008; Gatenby and Gillies, 2008; Gillies et al., 2012).

Hypoxia is broadly studied as a biologically and clinically important phenomenon with pronounced effects on tumor phenotype and cancer progression (Harris, 2002). It attracts more and more attention, and the knowledge that has accumulated over the past two decades is impressive (Ratcliffe, 2013). The clear connection between hypoxia and poor prognosis as well as the resistance to conventional treatment modalities has already led to the implementation of modified treatment regimens and new prognostic/predictive markers to clinical practice (Chitneni et al., 2011; Lin and Hahn, 2012). Despite diverse manifestations of hypoxia that can range from moderate to severe, acute to chronic, and intermittent to persistent, reduced oxygen availability generally stimulates a distinctive set of cellular adaptive processes that include a shift to glycolytic metabolism, slowed cell proliferation, diminished cell adhesion, increased migration and invasiveness, increased angiogenesis and other energy-saving and metastasis-enabling alterations (Semenza, 2012). These changes occur as a consequence of the massive remodeling of the transcriptional program activated by a heterodimeric transcription factor called hypoxia-inducible factor (HIF) composed of two subunits, of which the α subunit is sensitive to oxygen. Thus, HIF-α is generally missing in well-oxygenated cancer cells due to the negative control of its stability by the PHD-VHL pathway, although certain oncogenic events can mediate its stabilization even under normoxic conditions. In hypoxia, PHD-VHL effects are invalidated, HIF-α is stabilized and following entry to the nucleus and dimerization with the constitutive HIF-β subunit it can either turn on or elevate the transcription of a myriad of genes containing HIF-responsive elements (HREs) in the regulatory regions. A detailed description of the molecular mechanisms related to isoforms of HIF-α, regulation of its stability and transactivation ability can be found in many excellent papers (Kaelin and Ratcliffe, 2008; Lendahl et al., 2009) and will not be discussed in this review. Here it is important to note that proteins encoded by HIF-regulated genes (including glucose and lactate transporters, glycolytic enzymes, pro-angiogenic growth factors and receptors, ion transporters, etc.) execute the adaptive responses to hypoxia and are therefore active players in tumor progression. Moreover, some of them are clinically exploited as hypoxia-associated biomarkers and anticancer therapy targets (Wilson and Hay, 2011).

As mentioned above, hypoxia triggers a shift toward the glycolytic metabolism that allows for the sustained, albeit less efficient production of energy in conditions of reduced or absent oxygen, a substrate of oxidative phosphorylation. This is critical to the survival of hypoxic tumor cells. Hypoxia also selects inherently glycolytic cells developed through oncogenic events. Importantly, glycolysis not only generates energy but also facilitates the synthesis of biomass (e.g., nucleotides, amino acids, and lipids) required for the production of new cells during tumor expansion. Therefore, tumor cells rely on glycolysis even in the presence of oxygen (Vander Heiden et al., 2009; Schulze and Harris, 2012). Moreover, some tumor cells strongly depend on glutaminolysis, which can feed the mitochondrial TCA cycle and pentose phosphate pathway and thereby contribute to the synthesis of fatty acids, nonessential amino acids, and nucleosides. The excessive quantities of glutamine consumed and metabolized by cancer cells can result in the secretion of alanine and ammonium, which accumulate in the extracellular milieu (Levine and Puzio-Kuter, 2010).

Due to an oncogenic metabolism shared to a variable extent among respiration, glycolysis and glutaminolysis, tumor cells generate an excess of acidic metabolic products, including lactic acid, protons, and carbon dioxide. To counteract the cytosolic accumulation of these acidic metabolites and avoid prolonged intracellular acidosis, cells activate constituents of the pH regulating machinery, including ion exchangers and transporters, above the threshold of their normal activities and in some cases also redirect the transmembrane ion fluxes compared to normal cells (Parks et al., 2011). Many of these constituents and their regulators are pH sensitive molecules and are thus activated once the intracellular pH (pHi) reaches acidic values incompatible with the biosynthetic reactions and signaling processes (Alper, 2006; Odunewu and Fliegel, 2013). The purpose of their activation is to return pHi to slightly alkaline values favorable to cell survival and proliferation. Elimination of intracellular acidosis generally occurs through the export of lactate and protons and through the import of bicarbonate ions generated by the hydration of CO_2_ via mechanisms that are described in more detail in the text below. This however leads to pericellular acidosis that often persists in tumor microenvironment because the acidic metabolic waste cannot be effectively removed by the abnormal tumor vasculature (Raghunand et al., 2003).

**Figure 1:**
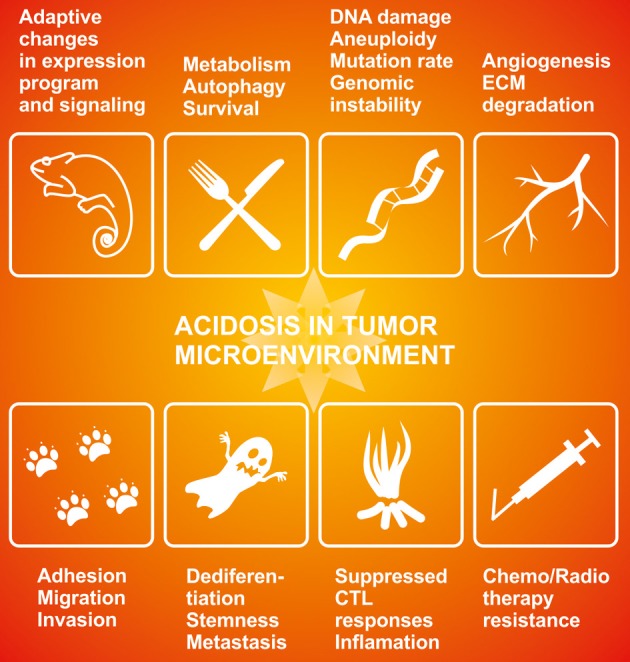
**The effects of acidosis on cancer-related phenomena**. Acidosis in tumor microenvironment influences key aspects of tumor phenotype and cancer behavior including expression reprogramming and signal transduction (Putney and Barber, 2004; Chen et al., 2008; Riemann et al., 2013), metabolism and survival (Parks et al., 2011; Marino et al., 2012; Wojtkowiak et al., 2012), genomic instability (Dai et al., 2013), angiogenesis (Shi et al., 2001; Peppicelli et al., 2013), adhesion-migration-invasion (Martínez-Zaguilán et al., 1996; Moellering et al., 2008; Stock and Schwab, 2009; Estrella et al., 2013), metastasis and stemness (Rofstad et al., 2006; Hjelmeland et al., 2011), aberrant immune responses (Fischer et al., 2000a,b; Mendler et al., 2012; Rajamäki et al., 2013), and therapy resistance (Thews et al., 2006; Wojtkowiak et al., 2011; Daniel et al., 2013).

Despite being less extensively studied than tumor hypoxia (and often remaining unappreciated as an inherent component of hypoxia), acidosis has similarly profound effects on tumor phenotype. Because tumor cells can adapt, acidosis endows them with the selective advantage against normal cells, which supports their expansion and dissemination. Thus, acidosis is associated with clinical phenomena such as chemo/radioresistance (Thews et al., 2006, 2010; Wojtkowiak et al., 2011), suppressed immune/CTL responses (Fischer et al., 2000a,b; Mendler et al., 2012), induced innate responses and inflammation (Rajamäki et al., 2013). It also supports progression-related phenomena such as angiogenesis (Shi et al., 2001), invasion (Martínez-Zaguilán et al., 1996; Moellering et al., 2008; Estrella et al., 2013), metastasis (Rofstad et al., 2006), and stemness (Hjelmeland et al., 2011). Finally, acidosis is linked with cellular phenomena including aneuploidy and mutation rate, autophagy and survival, cell migration, release of exosomes, etc. (Parolini et al., 2009; Stock and Schwab, 2009; Gillies et al., 2012; Marino et al., 2012; Wojtkowiak et al., 2012; Dai et al., 2013), see Figure 1. Although no master HIF-like regulator has been linked with acidosis, there are clear effects on activities of a number of transcription factors, as detected through gene profiling studies as well as through investigations of individual transactivators, such as SP1, HIF-1α, CREB and others (Torigoe et al., 2003; Mekhail et al., 2004; Shimokawa et al., 2005; Chen et al., 2008; Peppicelli et al., 2013; Riemann et al., 2013). This suggests that acidosis can influence the gene expression program of tumor cells.

## pH regulating machinery: a rescue apparatus of hypoxic/acidic tumor cells

Mechanisms of pH regulation in tumor cells are very complex and intertwined with other cancer-related processes. Put simply, pH-regulating machinery has two major arms as mentioned before: the lactate and proton export arm, and the bicarbonate import arm. The coordinated actions of these arms ensure that pHi is kept at neutral or slightly alkaline values (within narrow range of 7.2–7.4, which is similar for many tumor cell types), whereas extracellular pH (pHe) becomes acidic (ranging more broadly from 6.6 or even below to 6.9 in a tumor cell-related manner). The net result of this regulation is a reversed pH gradient, which affects ion fluxes, uptake of drugs, activation of proteases and other processes as mentioned above (Gerweck and Seetharaman, 1996; Pouysségur et al., 2006).

The export arm operates through several types of transmembrane ion transporters, represented in the cancer context mainly by proton-coupled monocarboxylate transporters (MCT4 and MCT1), by Na^+^/H^+^ exchanger 1 (NHE1), and vacuolar H^+^-APTase (Sennoune et al., 2004; Kennedy and Dewhirst, 2010; Reshkin et al., 2013). The import arm involves diverse bicarbonate transporters, including anion exchangers (AE1-3), electrogenic or electroneutral sodium-bicarbonate cotransporters (NBCe1, NBCn1), and related molecules (Lauritzen et al., 2010; Boedtkjer et al., 2013).

MCTs are 12-span transmembrane proteins encoded by the *SLC16A* gene family, which can transport lactate anion across the plasma membrane of tumor cells in association with proton (Halestrap, 2013). Of 14 MCT subtypes, only the first four isoforms MCT1-4 transport monocarboxylates like lactate, pyruvate, and ketone bodies in cotransport with proton. MCT1 (SLC16A1) and MCT4 (SLC16A3) are the two isoforms most relevant for cancer physiology. Their expression and transport activity are closely linked with the mature form of CD147 chaperone and in turn, expression of MCT1 and 4 is required for the maturation of CD147. All three molecules play active roles in tumor biology through their growth-promoting, pro-survival, and pro-metastatic effects (reviewed in Kennedy and Dewhirst, 2010). Both MCT1 and MCT4 can mediate lactate export as well as import, but MCT1 has a greater affinity for lactate than MCT4 (although the affinity of lactate in MCT4 can be increased by exposure to lower pH values). Moreover, MCT1 is expressed in most cells, whereas MCT4 is expressed strongly only in glycolytic tissues, which must export large amounts of lactic acid (e.g., white muscle and tumors). Thus, MCT4 primarily mediates lactate efflux from hyperglycolytic cells and is upregulated by hypoxia (Ullah et al., 2006). On the other hand, MCT1 is independent of hypoxia, activated in response to exogenous lactate, and can equally operate in both directions, enabling the export of intracellular lactate produced by glycolysis (in both anaerobic and aerobic conditions) as well as the import of extracellular lactate as a source of energy for the oxygenated cancer cells (Sonveaux et al., 2008).

Elevated lactate has been correlated with poor disease-free, metastasis-free and overall survival in various types of cancer (Walenta et al., 2000; Brizel et al., 2001; Walenta and Mueller-Klieser, 2004; Dhup et al., 2012). However, lactic acidosis in the absence of hypoxia appears to be rather a good prognostic factor in clinical breast cancer trials in contrast to hypoxia and non-lactate acidosis, which indicate poor prognosis (Chen et al., 2008). This is indeed compatible with the observations that low pHe cannot be primarily attributed to lactate or its MCTs-mediated transport and that acidification of tumor microenvironment can proceed in the absence of glycolysis as shown in studies of glycolysis-impaired or lactate dehydrogenase-deficient tumor cells (Newell et al., 1993; Yamagata et al., 1998; Helmlinger et al., 2002).

NHE1 (SLC9A1) can efficiently extrude intracellular protons in exchange for sodium ions. It is an important regulator of both pHe and pHi in tumors and contributes to the production and maintenance of a reverse proton gradient across the plasma membrane of tumor cells. It also participates in the regulatory volume increase, cytoskeletal anchoring and reorganization, cell migration, invasion, and other processes associated with tumor progression (Putney and Barber, 2004; Pedersen, 2006; Stock and Schwab, 2009). NHE1 is an 11-span integral membrane transport protein upregulated and/or activated during the oncogene-dependent transformation, as well as by various growth factors and hormones, by the extracellular matrix (ECM) receptor activation and by the physiological components of tumor microenvironment such as low serum, acidic pHe and hypoxia (Cardone et al., 2005; Shimoda et al., 2006; Boedtkjer et al., 2013; Reshkin et al., 2013, for more detailed overview see Harguindey et al., 2013). Activity of NHE1 is regulated by NHERF1 (Na^+^/H^+^ exchanger regulatory factor), a hypoxia and serum deprivation-induced PDZ domain-containing protein, which recruits membrane receptors/transporters and cytoplasmic signaling proteins into functional complexes. Through its PDZ domain NHERF1 coordinates a protein kinase A-gated RhoA/p38/NHE1 signaling and thereby enhances invasive phenotype of breast cancer cells (Cardone et al., 2007).

Importantly, recent studies have shown that proton extrusion by NHE1 represents a dynamic response to a larger acid load, which varies in a cell-specific manner and in most cells with high normoxic activity it is rather inhibited by hypoxia (Hulikova et al., 2013). Indeed, Na^+^/H^+^ exchanger is minimally active at physiological pHi and most active at a very acidic pHi around 6.6 (Lee and Tannock, 1998; Boedtkjer et al., 2013).

Thus, tumor cells dealing with a less acidic pHi close to the physiological value (i.e., between 7.2 and 6.9) stabilize their resting pHi at a mildly alkaline level preferentially through bicarbonate import by a family of bicarbonate transporters, such as Na^+^-coupled HCO^−^_3_ co-transporters NBCe1 (SLC4A4), NBCn1 (SLC4A7) and Cl^−^/HCO^−^_3_ anion exchangers such as AE2 (SLC4A2). Accordingly, NBCe1 is most active at a pH of around 6.9 (Lee and Tannock, 1998). Moreover, the sodium-coupled bicarbonate transport is a constitutive and stable element of pH regulation that can effectively proceed in hypoxia (Hulikova et al., 2013).

Bicarbonate transporters are widely distributed, expressed in diverse isoforms and splice variants, and play important roles in maintaining pHi as well as contributing to cell volume control, cell migration and other cancer-related phenomena (Alper, 2006; Boron et al., 2009).

Indeed, expression of several SLC4 family members was linked with cancer. Particularly NBCn1 (SLC4A7) is upregulated in breast cancer cells by ErB2/HER2 oncogene and its elevated expression correlates with poor prognosis of patients with mammary tumors (Lauritzen et al., 2010; Boedtkjer et al., 2013). Interestingly, bicarbonate transporters can functionally substitute NHE1 as demonstrated in NHE1 null mice, which induce AE2 activity in salivary exocrine cells to rescue pHi regulation (Gonzalez-Begne et al., 2007). Although anion exchangers are generally viewed as acid loaders, it is imaginable that this may change in situations where the gradient of substrate dictates an opposite direction of transport (such as in cancer cells or in lung erythrocytes, see Casey, 2006). In addition, pHi recovery from intracellular acidification in the hypoxic core cells of 3D tumor spheroids is slower upon inhibition of bicarbonate transport than upon inhibition of Na^+^/H^+^ exchange suggesting that bicarbonate transport is critical for coping with intracellular acidosis in tumor cells (Hulikova et al., 2013).

However, pericellular acidosis does not permit spontaneous formation and accumulation of bicarbonate ions as they would immediately dissipate in the acidic extracellular milieu. In fact, it has been shown that tumors contain less bicarbonate than normal tissues (Stubbs et al., 1994). Thus, in the absence of a catalytic facilitator locally producing bicarbonate ions close to the bicarbonate transporters, this lack of substrate would blunt or reduce the bicarbonate import and would not allow for the activation of this pH regulatory arm.

## CA IX joins the cast

Actually, this is the situation that brings the carbonic anhydrase IX (CA IX) to the scene. As a hypoxia-induced enzyme with an active site facing the extracellular space and an ability to efficiently catalyze the conversion of carbon dioxide to bicarbonate ions and protons, CA IX has important prerequisites to facilitate bicarbonate transport for the purpose of the intracellular alkalinization (Pastorekova et al., 2008). It is believed that CA IX uses its extracellular active site to catalyze CO_2_ hydration in a close spatial and functional cooperation with bicarbonate transporters (according to a so-called bicarbonate metabolon concept). The catalysis leads to the fast local production of bicarbonate ions that are directly delivered to bicarbonate transporters (such as NBCe1 and AE2), which bring them into the cytosol (Ditte et al., 2011; Svastova et al., 2012). There they can take up protons to generate CO_2_ that can leave the cell by diffusion and acidify the pericellular milieu (Swietach et al., 2008). The same CO_2_ hydration reaction catalyzed by CA IX generates also protons that remain on the outer side of the plasma membrane and further feed acidosis (Svastova et al., 2004). This model is supported by the experimental evidence of the *in vitro* interaction between the extracellular catalytic domain of CA IX and AE2 as well as between CA IX and NBCe1 (namely its extracellular loop 4) and also by the demonstration that CA IX accelerates the bicarbonate flux through these transporters (Morgan et al., 2007; Orlowski et al., 2012). Moreover, there is also *in situ* evidence obtained in living cells by a proximity ligation assay that CA IX co-localizes and interacts with bicarbonate transporters in the membranes of migrating cells, namely in their protruding areas that are characterized by intense ion transport (Svastova et al., 2012). Interestingly, *in vitro* measurements suggest that the CO_2_ hydration activity of CA IX is inhibited by an increasing concentration of bicarbonate ions but not by lactic acid, and that the pKa optimum of CA IX is around 6.5 (Innocenti et al., 2005, 2009). These data support the view that this enzyme is well equipped for the acidic tumor microenvironment.

**Figure 2:**
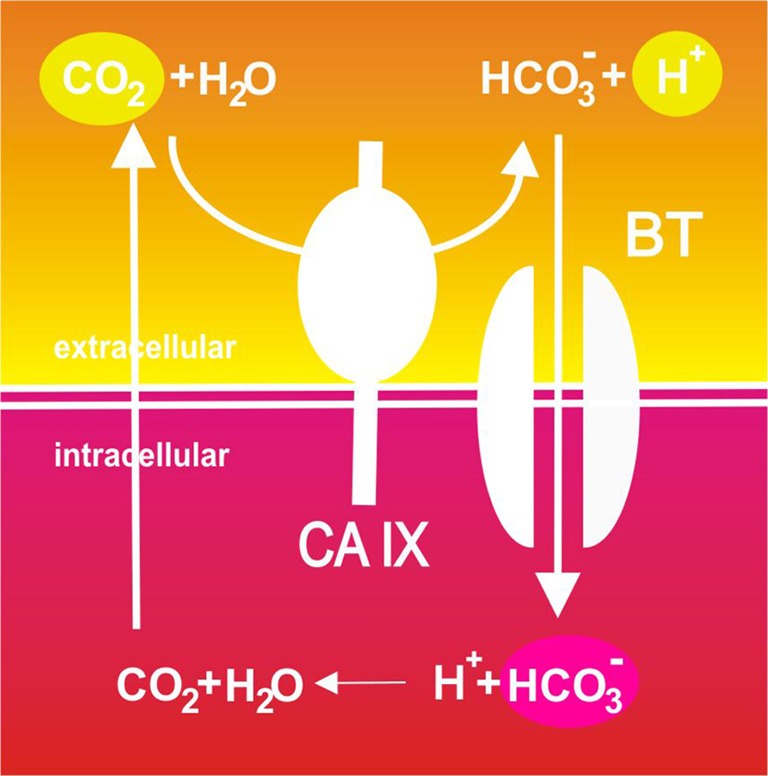
**Schematic illustration of the catalytic role of CA IX in pH regulation in tumor cells**. Pericellular CO_2_ is hydrated to bicarbonate ions and protons in a reaction efficiently catalyzed by the hypoxia-induced CA IX. Protons remain on the outer side of the plasma membrane and contribute to extracellular acidification. On the other hand, CA IX-facilitated production of bicarbonate ions is spatially and functionally coupled to their inward transport across the plasma membrane through bicarbonate transporters (BT) where these ions consume cytoplasmic protons and neutralize intracellular pH. This results in production of CO_2_ that leaves the cell by diffusion and may enter a new round of hydration.

So, depending on the angle of view and experimental approach, CA IX can act at three interdependent points of the so-called Jacob-Stewart cycle: it generates extracellular protons (to decrease pHe), provides bicarbonate ions for transport and intracellular consumption of protons (to increase pHi) and facilitates CO_2_ diffusion (to maintain pHi>pHe gradient), see Figure 2. Moreover, it has also been suggested that CA IX cooperates with proton exporters by providing bicarbonate ions for taking up a fraction of protons extruded from cells and facilitating their extracellular mobility throughout tumor tissue (Hulikova et al., 2012). This may be particularly important at a very low pHe, when transmembrane bicarbonate fluxes are inhibited and bicarbonate ions generated by the catalytic activity of CA IX cannot be imported to the cytoplasm. Thus, CA IX appears to spatially coordinate ion fluxes and pH regulation in tumor tissues.

This begs the questions as to which circumstances and molecular mechanisms make CA IX such an important pH regulator, and which are the situations relevant for tumor biology that involve the enzyme activity or other molecular attributes of CA IX.

Before answering these questions, it is important to note that CA IX is one of the 15 human isoforms of the α carbonic anhydrase family (Pastorek et al., 1994), members of which are well-known players in normal physiological processes that require intense ion transport and pH regulation in virtually all cells/tissues of the human body. They are localized in various subcellular compartments and through their catalytic activity in the reversible hydration of carbon dioxide ranging from no/low to high are involved in the production of gases, body fluids, bone resorption, biosynthetic reactions etc. (Pastorekova et al., 2004). With the exception of CA IX, they are all predominantly expressed in differentiated tissues and some isoforms are also present in certain tumors, including CA II in GIST and endothelium of brain tumors and CA XII in kidney, breast cancer etc. (Kivelä et al., 2000; Parkkila et al., 2000, 2010; Haapasalo et al., 2008; Korhonen et al., 2009). In contrast, CA IX is absent from the majority of normal tissues (being abundant only in the stomach and gallbladder epithelia). However, it is very often and strongly expressed in tumors, generally in their more aggressive variants and growing clinical evidence suggests that it can serve as a prognostic indicator and/or predictive factor, and a therapeutic target (reviewed in Pastorek and Pastorekova, 2010; Oosterwijk-Wakka et al., 2013). Thus, CA IX tissue distribution itself offers an argument that it is actually needed in tumor cells.

## Regulation of CA IX expression and function: is it all about hypoxia?

CA IX is one of the best responders to hypoxia (ranging from anoxia to moderately reduced oxygen), in some cell types achieving several dozen or hundred-times induction. This is mainly because the CA IX-encoding gene (according to the nomenclature designated *CA9*) is transcriptionally regulated by hypoxia through HIF-1 binding to an HRE sequence localized immediately in front of the transcription initiation site (Wykoff et al., 2000). The HRE is thus a critical component of the core promoter of the *CA9* gene together with an adjacent SP1-binding site. This SP1-HRE module is a principal mediator of the *CA9* transcription, albeit additional cis-elements localized 5′ to the core promoter can have modulatory effects (Kaluz et al., 2002). SP1 appears to play a role in the induction of the *CA9* transcription by an increased cell density and presumably also by an acidosis (both normoxic and hypoxic), in a cell type-specific manner possibly depending on a threshold capacity of a given cell type to regulate pH (Kaluz et al., 2002; Ihnatko et al., 2006). Moreover, since expression and activation of HIF are affected by oncogenic signaling, transcription of the *CA9* gene also increases in response to activation of the MAPK and PI3K pathways and upstream tyrosine kinases, including SRC oncoprotein, EGFR (epidermal growth factor receptor) and RET (Rearranged during Transfection) oncoprotein, as well as to viral oncoproteins, such as HBx (Kopacek et al., 2005; Holotnakova et al., 2010; Takacova et al., 2010). Expectedly, genetic inactivation of the pVHL tumor suppressor protein, which negatively controls HIF stability, also results in the elevation of CA IX in kidney tumors (Ivanov et al., 1998; Wykoff et al., 2000; Stillebroer et al., 2010).

Hypoxia also regulates the splicing of CA IX mRNA (Barathova et al., 2008), and activates CA IX protein at the functional level through the hypoxia-induced increase in cAMP levels and activation of the protein kinase A, which phosphorylates Thr443 in the intracellular tail of CA IX (Ditte et al., 2011). Phosphorylation of Thr443 then mediates signaling to extracellular catalytic domain that leads to activation of the CA IX capacity to regulate pH. CA IX is a very stable protein of about 40 h half-life and hypoxia can induce the cleavage and release of its ectodomain (ECD) from the cell surface to the extracellular space, which is executed by TACE/ADAM17 that is itself regulated by hypoxia (Rafajova et al., 2004; Zatovicova et al., 2005). The role of the CA IX ECD remains to be elucidated. However, since all known TACE substrates are not decoys but function as biologically active molecules, we believe that the CA IX ECD mediates autocrine and/or paracrine intercellular signaling.

Moreover, in certain cell types and conditions, hypoxia can induce internalization of CA IX subpopulation and its recycling through the endosomal compartment (Svastova et al., 2003; Zatovicova et al., 2010). Endocytosis is an important process that affects stability and activity of regulatory molecules and has many effects on signal transduction. It involves invagination of plasma membrane regions with resident proteins and formation of endosomes that further carry these proteins either to lysosomal compartment for degradation or to recycling compartment for their return to the cell surface. Thereby endocytosis can either attenuate or prolong the receptor-mediated signaling (Sorkin and von Zastrow, 2009). It remains to be elucidated whether CA IX can signal from endosomes and whether endocytosis of CA IX is relevant for tumor phenotype. Despite shedding and endocytosis can reduce the plasma membrane level of CA IX, hypoxia strongly induces expression of new CA IX molecules, so these processes cooperate with each other in modulation of the CA IX abundance (explained in more detail in Zatovicova and Pastorekova, 2013). Thus, it is quite apparent that hypoxia influences many steps in the CA IX biogenesis, transport and functioning.

Because extracellular acidosis is an inherent component of hypoxia that often remains unrecognized, it is difficult to discriminate its contribution to each of the above-mentioned processes regulating expression and function of CA IX. Indeed, data available in the literature show inconsistent and contradictory effects of acidosis on CA IX expression (Ihnatko et al., 2006; Willam et al., 2006; Sørensen et al., 2007). Since the large genomic studies show remarkable differences in expression profiles and their prognostic values in hypoxia, lactic acidosis, and normoxic acidosis, it is very important to distinguish between these situations, which can concurrently occur in the heterogeneous tumor tissue (Chen et al., 2008). One possible approach toward the study of the impact of hypoxic acidosis includes buffering of the extracellular pH in hypoxic conditions. Initial data from our experiments indicated that buffering of hypoxic acidosis leads to reduced CA IX mRNA levels even in the cell lines that did not show marked CA IX induction by the normoxic acidosis. This is in agreement with the recent observation that CA IX expression in osteosarcoma cell lines was highest in the combination of acidic (pH 6.8) and hypoxic growth conditions (Matsubara et al., 2013).

Immunohistochemical data related to CA IX staining pattern in tumor tissue clearly reflect the complex, but hypoxia-dominating regulation of the CA IX expression. In renal cell carcinomas, particularly of the clear cell type (CCRCC), which often develop due to the genetic inactivation of the *VHL* gene, HIF is also stabilized and activated in the absence of physiological hypoxia (Wiesener et al., 2001). Therefore, CA IX is overexpressed in more than 90% of these CCRCC tumors (Stillebroer et al., 2010; Takacova et al., 2013). Expression of CA IX can be detected in a high percentage of tumor cells and despite showing a tendency to decrease at more advanced CCRCC stages, the cut-off for positivity is 85% of cells in the tumor tissue, suggesting that CA IX level is still very high (Bui et al., 2003). In many other tumor types carrying the wild-type *VHL* gene, CA IX expression exhibits typical focal hypoxic pattern (Wykoff et al., 2000). It is often present in a broader perinecrotic area corresponding to the fact that it can be transactivated already at moderate hypoxia, mainly in the chronic setting. Thus, CA IX is also expressed in moderately hypoxic viable tumor cells that are adaptable to low oxygen and possess strong metastatic potential. Due to this responsiveness to moderately reduced oxygen level, CA IX distribution only partially overlaps with the distribution of HIF-1α as well as of additional hypoxia-induced proteins with different hypoxic thresholds for induction (Wykoff et al., 2000; Hui et al., 2002; Swinson et al., 2004). Sometimes, the CA IX expression pattern is rather diffuse, possibly reflecting oncogenic alterations. Moreover, CA IX can occasionally be found in HIF-1α-negative areas, possibly because reoxygenation leads to degradation of HIF-1α, but not of the highly stable CA IX protein. On the other hand, the absence of CA IX in HIF-1α positive areas may indicate increased shedding of the CA IX ectodomain. Of course, many other factors, including microenvironmental acidosis may influence the CA IX distribution. Indeed, acidosis can elevate the activities of proteases, modulate cross-talk with the ECM components, increase the release of exosomes and modulate the local response to hypoxia (Raghunand et al., 2003; Daniel et al., 2013). So the incomplete overlap of CA IX with HIF-1α may at least to some extent mirror the discordant relationship between profiles of the partial pressure of oxygen and local pH profiles in tumor tissue, resulting from a heterogeneity in regional pH and pO_2_ distribution patterns and absence of their spatial correlation (Helmlinger et al., 1997).

**Figure 3:**
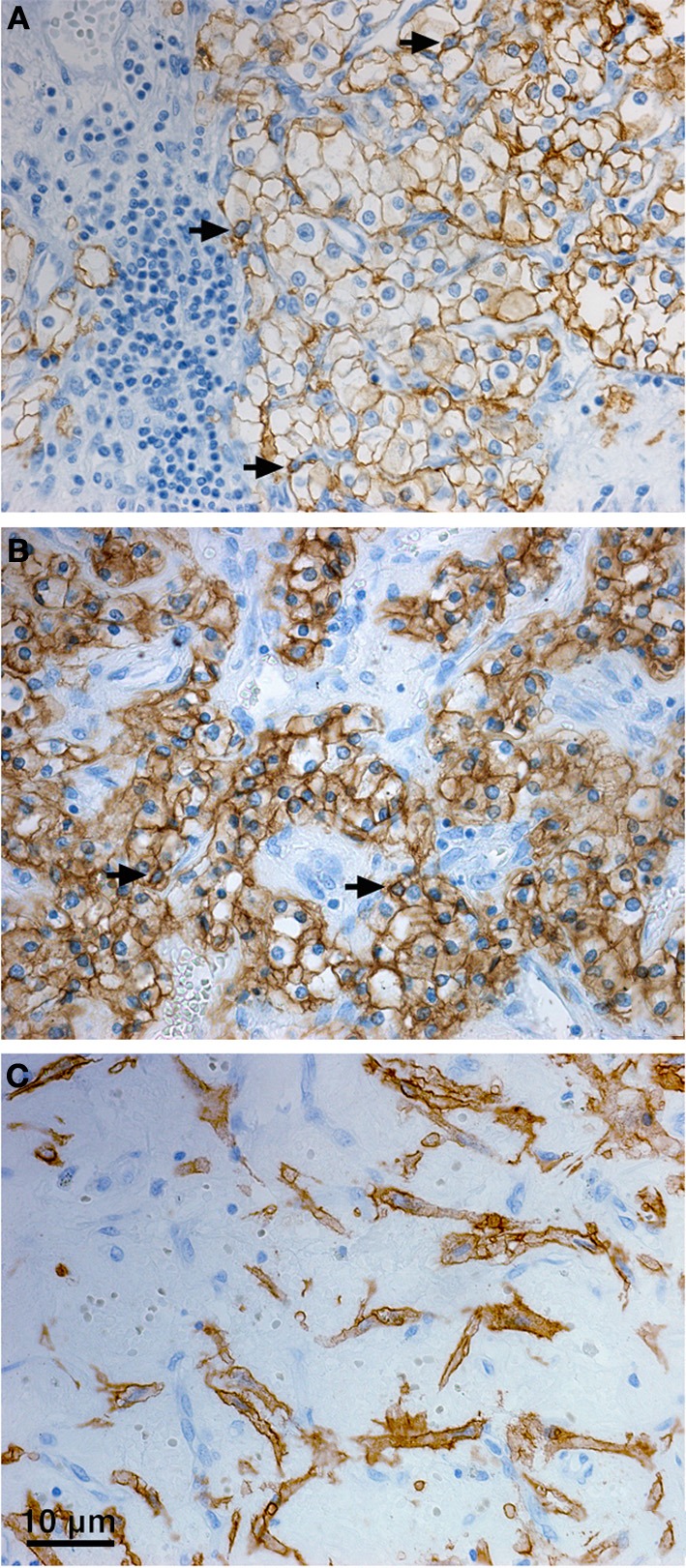
**Distribution of CA IX in the tumor tissue specimen from CCRCC patients**. Immunohistochemical staining pattern of CA IX shows positive staining signal predominantly localized in the plasma membranes **(A)**, but also in cytoplasm **(B)**, and perinuclear regions (arrows, A, B) of the tumor cells. Moreover, CA IX can be detected in the stromal cells **(C)**. Tissue processing and staining procedures were as described in Takacova et al. (2013). Briefly, dissected tissues were embedded in paraffin according to the standard histological procedure and sectioned. Immunohistochemistry was performed on an automated immunostainer (Dako) using M75 antibody (hybridoma medium diluted 1:100) and DakoCytomation Envision® + System-HRP according to the manufacturer's instructions. Staining was visualized with DAB solution for 1 min with 3,3′-diaminobenzidine as a chromogenic substrate. Finally, the stained sections were counterstained with Mayer's hematoxylin, mounted, and examined.

With regard to the subcellular localization, CA IX is a type I transmembrane protein and thus it is mostly stained at the plasma membrane of tumor cells. Although the plasma membrane is the main site of residence of the mature CA IX protein and several studies have shown its prognostic value, there are also other compartments where CA IX can be found by immunodetection methods in cultured cells as well as in tumor tissues. The cytoplasmic staining signal is often seen in tumor sections (sometimes very strong, see Figure 3), but it is generally ignored despite the known fact that CA IX can be endocytosed and that cell receptors can signal not only from the cell surface but also from endosomes (Joffre et al., 2011; Zatovicova and Pastorekova, 2013). So, the intracellular localization of CA IX may have its biological meaning as explained above. CA IX can be also found in the cellular components of tumor stroma (Figure 3). This stromal signal represents either induction of endogenous CA IX in cancer-associated fibroblasts (Fiaschi et al., 2013), or CA IX ECD released from tumor cells and bound to the surface of immune cells or potentially endocytosed in these cells (Wang et al., 2008). It has actually been shown that CA IX ECD binds to the monocyte-derived dendritic cells, so this option cannot be excluded for other immune cell types either. Finally, in agreement with the experimental evidence for the CA IX shedding, the CA IX ECD can be detected in the body fluids of cancer patients (Závada et al., 2003; Hyrsl et al., 2009; Ilie et al., 2010; Zhou et al., 2010; Wind et al., 2011; Schütze et al., 2013). This fact can be clinically exploited for the non-invasive screening or monitoring of cancer patients.

## CA IX role in cancer biology: is it all about pH regulation?

**Figure 4:**
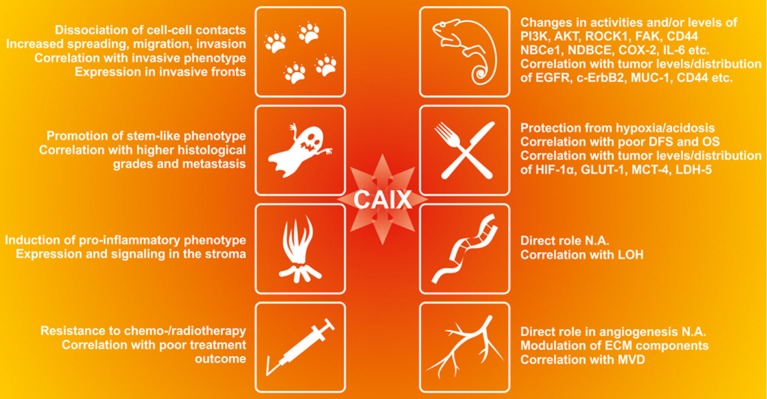
**The functional contribution of CA IX to the cancer-related phenomena affected by acidosis in tumor microenvironment**. CA IX appears to be involved in many aspects of tumor progression as supported by the experimental evidence in preclinical models as well as by the clinical data from tumor tissue specimens. CA IX induces changes in activities and/or levels of kinases, ion transporters and other regulatory molecules and its expression correlates with certain cancer markers (Giatromanolaki et al., 2001; Bartosova et al., 2002; Swinson et al., 2004; Dorai et al., 2005; Skrzypski et al., 2008; Shin et al., 2011; Csaderova et al., 2013; Radvak et al., 2013), CA IX protects tumor cells from hypoxia and acidosis and its expression correlates with poor disease-free survival (DFS), overall survival (OS) and markers of tumor hypoxia and metabolism (Beasley et al., 2001; Koukourakis et al., 2006a,b; Chiche et al., 2009; Rajaganeshan et al., 2009; Lou et al., 2011; Rademakers et al., 2011; McIntyre et al., 2012; Kim et al., 2013). Although its direct role in genomic instability and angiogenesis has not been analyzed (N.A.), CA IX modulates expression of EMC components including MMP2, MMP9 and collagen IV (Radvak et al., 2013) and its expression correlates with loss of heterozygozity (LOH) in head and neck carcinoma (De Schutter et al., 2006) and with microvascular density (Couvelard et al., 2005a,b). CA IX also reduces cell-cell adhesion, increases migration and invasion of tumor cells (Svastova et al., 2003; Sansone et al., 2009; Svastova et al., 2012) and its expression and distribution correlates with invasive tumor phenotype (Chen et al., 2005; Rajaganeshan et al., 2009; Deschamps et al., 2012);. CA IX also promotes stemness and correlates with higher histological grades and metastatic disease (Sansone et al., 2007a,b; Storci et al., 2008; Chen et al., 2010; Currie et al., 2013; Fujiwara et al., 2013; Lock et al., 2013). CA IX induces pro-inflammatory phenotype and participates in communication between cancer cells and stroma (Sansone et al., 2009; Fiaschi et al., 2013). It is associated with increased resistance to conventional therapy and its expression correlates with poor therapy outcome (Tomes et al., 2003; Cleven et al., 2007; Brockton et al., 2011, 2012; Guedj et al., 2011; Kwon et al., 2013).

CA IX expression represents a strong and frequent adaptive response to hypoxia and/or acidosis, indicating that it is beneficial for cancer cells exposed to these microenvironmental stresses. Both experimental and clinical evidence shows that CA IX can confer several selective advantages, particularly in situations associated with the phenotypic consequences of a disturbed pH homeostasis (Figure 4).

Firstly, CA IX provides a survival advantage in hypoxia and/or acidosis. This was clearly demonstrated in several studies using various preclinical models with genetically manipulated CA IX expression (Chiche et al., 2009; McIntyre et al., 2012) or using inhibitors of the CA activity (Lou et al., 2011). In all cases, CA IX suppression, deletion of the catalytic domain, or pharmacologic inhibition resulted in significantly reduced growth of tumor xenografts. It is quite apparent that tumor cells lacking CA IX or its enzyme activity were unable to cope with the deleterious effects of microenvironmental stress. In support of this assumption, shRNA silencing causing CA IX-deficiency in HT1080 tumor cells led in decreased extracellular acidosis in hypoxia (Radvak et al., 2013). Moreover, clinical data based on immunohistochemistry of various tumor types show that CA IX is frequently associated with cancer progression and poor prognosis and in some tumor tissues it is co-expressed with the markers of oncogenic metabolism and acidosis, such as LDH5, GLUT1, MCT4 etc. (Beasley et al., 2001; Koukourakis et al., 2006a,b; Rajaganeshan et al., 2009; Rademakers et al., 2011; Kim et al., 2013).

Secondly, CA IX appears to confer resistance to therapy, including chemotherapy, radiotherapy, and anti-angiogenic treatment. The preclinical evidence again comes from the studies of animals xenografted with human tumor cells differing in expression of CA IX and treated with radiation or various drugs (chemotherapeutic agents and CA inhibitors). Tumors with a high CA IX level were less responsive to experimental therapy, but inhibition of CA IX catalytic activity significantly improved their chemo- or radiosensitivity (Dubois et al., 2011, 2013). Similarly, CA IX inhibition enhanced the effect of anti-angiogenic therapy with anti-VEGF antibody (McIntyre et al., 2012). These findings suggests that the pH regulatory function of CA IX is important for the protection of tumor cells from the toxic effects of drugs or radiation, in agreement with the concept that extracellular acidosis may negatively affect drug uptake and radiation damage (Wojtkowiak et al., 2011). There are also many clinical data demonstrating the correlation between CA IX expression and poor therapy outcome in cancer patients (Giatromanolaki et al., 2001; Span et al., 2003; Koukourakis et al., 2006a,b, 2008; Korkeila et al., 2009; Tan et al., 2009).

**Figure 5:**
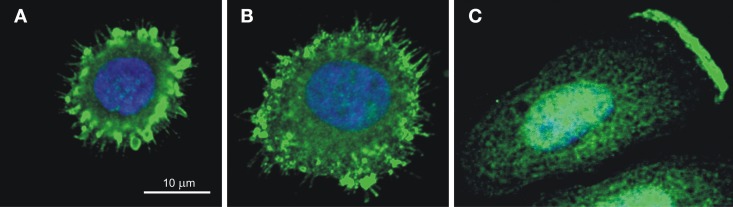
**CA IX localization in the spreading cells (A,B) and in the lamellipodium of the migrating cell (C)**. Immunofluorescence staining of hypoxic SiHa cells attaching to the collagen support shows that CA IX is recruited **(A)** to filopodia and to emerging focal contacts in the early stage of spreading (10 min after seeding) and **(B)** to maturing focal adhesions in the later stage (20 min after seeding). SiHa cells incubated in hypoxia for 48 h and stimulated to migration by hepatocyte growth factor for 2 h exhibit the CA IX signal concentrated on the protruding cell front, in the plasma membrane of the lamellipodium **(C)**. The CA IX staining was performed using M75 monoclonal antibody followed by anti-mouse IgG conjugated with Alexa Fluor® 488 and the nuclei were stained with DAPI as described in Svastova et al. (2012) and Csaderova et al. (2013).

CA IX also facilitates the migration and invasion of tumor cells through participation in pH regulating apparatus of the lamellipodial membranes at the protruding fronts of moving cells (Svastova et al., 2012). Migration and invasion are the initial steps of the metastatic cascade that can be stimulated by hypoxia as well as by extracellular acidosis, which activates growth factors and extracellular matrix-degrading proteases. The ability of cells to migrate strongly depends on the establishment of the correct pH gradients along the longitudinal cell axis, with acidic pHe and alkaline pHi at the cell front, and alkaline pHe and acidic pHi at the cell rear (Stock and Schwab, 2009; Martin et al., 2011). This is connected with the accumulation of an array of ion transporters and pumps in the lamellipodial membranes accomplished through a directed vesicular transport that includes endocytosis and recycling (Schwab et al., 2012). CA IX is also relocalized to the lamellipodia of migrating cells (see Figure 5), where it was shown to interact with bicarbonate transporters in order to facilitate bicarbonate import and maintain optimal pH gradient as discussed in more detail elsewhere (Svastova et al., 2012; Svastova and Pastorekova, 2013). Importantly, this function of CA IX requires intact catalytic domain suggesting that it is related to pH regulation. Moreover, CA IX participates in the formation of nascent adhesion contacts at the leading edge of migrating cells, where these dynamic structures mediate transmission of large forces during the forward movement of the cell body (Csaderova et al., 2013).

In addition, CA IX contributes to the assembly and maturation of focal adhesion contacts during initial cell spreading (Csaderova et al., 2013), Figure 5. Interestingly, cells expressing the truncated form of CA IX lacking the N-terminal PG-domain as well as the cells expressing the full length CA IX protein and treated with the PG domain-binding M75 antibody display a significantly reduced spreading (Csaderova et al., 2013). The PG domain exhibits an amino-acid sequence homology to proteoglycans and is absent from the other CA isoenzymes (Opavský et al., 1996). Although *in vitro* measurements indicate that the PG domain can stimulate the catalytic activity of CA IX, its deletion did not affect the ability of CA IX to acidify extracellular medium in hypoxic MDCK cells (Svastova et al., 2004; Innocenti et al., 2009). Thus, it is difficult to judge whether or not the CA IX role in cell spreading is related to its pH regulating function and more experimentation is needed to come to a reliable conclusion. Moreover, while pH nanodomains were recognized in lamellipodial focal adhesions of migrating cells (Ludwig et al., 2013), no such phenomenon has so far been described for focal adhesion contacts of spreading cells, urging further investigations in this direction.

CA IX also appears to be involved in the destabilization of cell-cell adhesion contacts, which is the first prerequisite for the detachment of cells from the tumor tissue and their morphological remodeling toward the migration-prone mesenchymal phenotype (Svastova et al., 2003). The underlying mechanism includes the CA IX-mediated disconnection of E-cadherin from the cytoskeletal anchorage through the competitive binding to beta catenin (Svastova et al., 2003). Again, it is not clear whether this propensity of CA IX relates to its pH regulating function. In fact, there are almost no data related to pH effects on E-cadherin expression and/or its function during tumor progression. However, acidosis in the synaptic cleft reduced the N-cadherin-mediated adhesion and facilitated synaptic remodeling (Baumgartner et al., 2013), so it is imaginable that a similar situation in tumor tissue may influence adhesion between tumor cells.

Experimental evidence for the role of CA IX in cell migration and invasion includes the CA IX suppression in HT1080 cells leading to reduced spreading and migration (Radvak et al., 2013), CA IX suppression in colorectal carcinoma cells leading to reduced invasion capacity in a COX-2 related manner (Sansone et al., 2009), as well as the pharmacologic inhibition of CA IX in mice xenografted with breast cancer cells leading to reduced growth of metastases (Gieling et al., 2012). There are also a number of clinical studies showing the correlation between the CA IX expression and invasive phenotype (with CA IX localized at the invasive front) or metastatic propensity (Chen et al., 2005, 2010; Kon-no et al., 2006; Rajaganeshan et al., 2009).

Increased expression of CA IX at advanced stages/grades of many tumor types also suggests its association with dedifferentiation. Hypoxia is one of the driving forces of cancer cell dedifferentiation toward the stem cell-like phenotype (Axelson et al., 2005). This phenotypic remodeling occurs through upregulation of molecules contributing to stemness and CA IX appears to be involved in this phenomenon (Sansone et al., 2007a,b; Storci et al., 2008; Fujiwara et al., 2013). Its expression correlates with the expression of a stem cell marker CD44 in breast carcinomas (Chen et al., 2010; Currie et al., 2013; Fujiwara et al., 2013) and its targeting results in the inhibition of breast cancer stem cell expansion in hypoxia (Lock et al., 2013). Since the depletion of breast cancer stem cells was observed upon the inhibition of the CA IX catalytic activity, we may propose that the pH-regulating function of CA IX is required for the maintenance of stemness. This is indeed the indication of the importance of pH regulation for cancer stem cell phenotype, in line with the data from literature relating extracellular acidosis to the promotion of stemness (Hjelmeland et al., 2011).

Finally, CA IX appears to be involved in communication between tumor cells and cellular components of the stroma. It has recently been demonstrated that CA IX expression can be induced in cancer-associated fibroblasts (CAFs) in response to ROS-mediated stabilization of HIF-1 in normoxia and that up-regulation of CA IX in CAFs leads to extracellular acidosis and activation of EMT in epithelial cancer cells. Importantly, the silencing of CA IX in CAFs is sufficient to prevent lung metastasis from co-injected prostate cancer cells *in vivo*, suggesting its crucial role in cancer progression (Fiaschi et al., 2013). In addition, earlier studies suggest that CA IX ectodomain can mediate paracrine signaling via binding to the surface of dendritic cells (DC) and potentially modulate their immune responses (Wang et al., 2008). This might be particularly interesting in light of the fact that hypoxia promotes recruitment and pro-inflammatory phenotype in DCs within tumor tissue (Bosco and Varesio, 2012). Again, clinical evidence from human tumors shows that stromal CA IX expression is strongly associated with the poor survival of head and neck cancer patients as well as in patients with colorectal cancer (Tomes et al., 2003; Cleven et al., 2007; Brockton et al., 2011, 2012; Kwon et al., 2013), and that in colorectal carcinoma, CA IX is co-expressed with the pro-inflammatory enzyme COX-2 (Sansone et al., 2009).

However, CA IX is not only an enzyme involved in pH control, but also a regulatory molecule involved in signal transduction. There are several studies exploiting mutagenesis or manipulated expression approaches, which link CA IX to major signal transduction pathways. These studies show that EGF-stimulated phosphorylation of Tyr449 of CA IX leads to the activation of PI3K/Akt pathway (Dorai et al., 2005), that Thr443 of CA IX transmits signals from the hypoxia activated PKA to stimulate bicarbonate production coupled with import (Ditte et al., 2011), that overexpression of CA IX promotes the activation of FAK/PI3K/mTOR/p70S6K pathway (Shin et al., 2011), and that CA IX deficiency reduces the activity of ROCK1 and affects expression of many migration-related genes including MMP2 and MMP9 (Csaderova et al., 2013; Radvak et al., 2013). These signaling events then lead to the phenotypic effects described above. Nevertheless, many other molecules, which transcriptionally respond to changes in CA IX levels, are listed in the microarray datasheets and their further investigation will surely shed more light on the position of CA IX in the signal transduction network. Immunohistochemical studies of various tumor tissues revealed that CA IX is co-expressed with diverse signaling molecules, including EGFR, MUC-1, cErb22, CD44, VEGF, etc. and at least in some cases this may suggest cross-talk between the pathways related to these molecules (Giatromanolaki et al., 2001; Bartosova et al., 2002; Swinson et al., 2004; Skrzypski et al., 2008).

## Conclusion

The data summarized in this overview clearly show that CA IX is functionally relevant for tumor phenotype particularly in connection with hypoxia and acidosis and that its biological role can be mainly attributed to pH regulation. In addition, there are certain situations, in which CA IX appears to act through the structural domains outside of the catalytic site and these actions appear to be pH-independent, at least at the first glance. However, many cellular processes have not been explored in detail in relation to pH nano- or microenvironments present in the subcellular compartments and to pH nano- or microdomains of the plasma membrane regions responding to various extracellular and intracellular stimuli (cell spreading, dissociation of cell-cell contacts, intracellular signaling, ectodomain signaling etc.). Thus, additional investigations are needed to elucidate whether these seemingly pH-unrelated features of CA IX are involved in cellular adaptations to dynamic pH perturbations or whether they contribute to different aspects of tumor biology.

### Conflict of interest statement

Jaromir Pastorek and Silvia Pastorekova are inventors of patents related to CA IX. The other authors declare that the research was conducted in the absence of any commercial or financial relationships that could be construed as a potential conflict of interest.

## Abbreviations

AE2: anion exchanger 2
ADAM: a disintegrin and metalloprotease
CA IX: carbonic anhydrase protein isoform 9
*CA9*: human carbonic anhydrase 9 gene
CAF: cancer-associated fibroblasts
CCRCC: clear cell renal cell carcinoma
COX: cyclooxygenase
CREB: cAMP response element binding
DC: dendritic cells
ECD: ectodomain
ECM: extracellular matrix
EGF: epidermal growth factor
EGFR: EGF receptor
EMT: epithelial-mesenchymal transition
ER: endoplasmic reticulum
FAK: focal adhesion kinase
GIST: gastrointestinal stromal tumor
GLUT: glucose transporter
HIF: hypoxia inducible factor
HRE: hypoxia-response element
LDH: lactate dehydrogenase
MAPK: mitogen activated protein kinase
MCT: monocarboxylate transporter
MMP: matrix metalloproteinase
MUC: mucin
NBCe1: electrogenic sodium-bicarbonate cotransporter
NHE: sodium-hydrogen exchanger
NHERF1: Na^+^/H^+^ exchanger regulatory factor 1
PHD: prolyl hydroxylases
pHi: intracellular pH
pHe: extracellular pH
PI3K: phosphatidyl inositol 3-kinase
PKA: protein kinase A
ROS: reactive oxygen species
SP1: specificity protein 1
TACE: TNFα converting enzyme
TNFα: tumor necrosis factor α
VEGF: vascular endothelial growth factor
VHL: von Hippel Lindau.
